# Mapping the research landscape of immune response in human brucellosis: a bibliometric analysis

**DOI:** 10.3389/fmicb.2025.1583520

**Published:** 2025-07-29

**Authors:** Lingling Wang, Peipei Lu, Xuhong Wang, Xue Song, Xuewei Tong, Shuting Yang, Zhiwei Li

**Affiliations:** Clinical Laboratory Center, People’s Hospital of Xinjiang Uygur Autonomous Region, Ürümqi, China

**Keywords:** human brucellosis, immune response, bibliometric analysis, type IV secretion system, vaccine development

## Abstract

**Introduction:**

*Brucella* bacteria are adept at evading the human immune system, leading to a chronic infectious disease known as brucellosis, which poses significant global health challenges. This study addresses a notable gap in bibliometric analyses concerning the immune response to brucellosis.

**Methods:**

We conducted a comprehensive literature screening from the Web of Science Core Collection, covering publications from 1980 to 2024. Relevant publications were analyzed using R software, VOSviewer, and CiteSpace for bibliometric analysis.

**Results:**

A total of 733 publications were included in this study, revealing an average annual growth rate of 5.91% in publications. The United States led in publication volume and citations, followed by China and France. Prominent research institutions included INSERM (France) and CONICET (Argentina). *Infection and Immunity* was identified as a leading journal in the field, publishing 97 papers with 5,184 citations. Keyword co-occurrence analysis delineated three main research clusters: *Brucella* vaccine development and immune protection, *Brucella* molecular mechanisms and intracellular survival strategies, and host innate immunity and *Brucella* interaction mechanisms. Burst analysis highlighted increasing attention on keywords such as “protection,” “type IV secretion system,” “pathogenesis,” and “prediction” since 2018.

**Discussion:**

This bibliometric analysis sheds light on the global research landscape regarding the immune response in human brucellosis, pinpointing trends and gaps, with a focus on immune escape mechanisms and the future development of safe, effective vaccines.

## Introduction

Brucellosis, caused by *Brucella* species, represents a significant global health challenge, with over 500,000 new cases reported annually. Among the 10 recognized *Brucella* species, four are pathogenic to humans, underscoring its prevalence as a zoonotic threat (Deng et al., 2019; Hull and Schumaker, 2018; Qureshi et al., 2023). In humans, brucellosis typically manifests as prolonged fever, sweating, joint pain, and hepatosplenomegaly. Severe cases can result in multi-organ damage and a significantly diminished quality of life (Zheng et al., 2018). Up to 30% of cases may progress to a chronic stage characterized by nonspecific symptoms and complications such as spondylitis and chronic fatigue syndrome, along with high relapse rates (Mitroulis et al., 2022). Recent study suggested that the incidence may be higher than previously estimated, with 1.6 to 2.1 million new cases occurring each year (Laine et al., 2023). Brucellosis remains prevalent in regions such as the Middle East, the Mediterranean, Central and South America, and East Asia (Khurana et al., 2021; Pappas et al., 2006).

The innate and adaptive branches of the immune system are essential for initiating and progressing diseases. Innate immunity serves as the first line of defense against danger signals in a rapid and nonspecific manner (Lizhe et al., 2020). Protection against *Brucella* and the prevention of its intracellular parasitism in macrophages depend on cell-mediated immunity, which necessitates a robust Th1 immune response and significant production of interferon-gamma (IFN-γ) (Skendros and Boura, 2013). Furthermore, innate immunity and neutrophils play pivotal roles in early proinflammatory response against *Brucella* (Moreno and Barquero-Calvo, 2020). However, *Brucella* has evolved various strategies to evade both innate and adaptive immune response, facilitating its establishment of long-term intracellular survival and replication (Martirosyan et al., 2011; Jiao et al., 2021). These immune evasion mechanisms may result from antigenic variation and intracellular parasitism (Mitroulis et al., 2022). Nonetheless, the immune response of human brucellosis remains incompletely understood, and integrated molecular data elucidating the complex interactions between *Brucella* and host immunity are currently lacking. Most related studies were traditional narrative reviews, deficient in quantitative and visualized macro-analyses (Byndloss and Tsolis, 2016). Understanding these mechanisms is critical for the development of novel therapeutic approaches and preventive strategies, including vaccines, aimed at reducing the global impact of brucellosis (Heidary et al., 2022).

Bibliometric analysis serves as a robust method for the exploration and analysis of extensive scientific data. It facilitates a comprehensive understanding of the evolutionary trends within a specific field and aids in the identification of emerging research directions (Donthu et al., 2021). Despite ongoing research on human brucellosis, comprehensive analyses of global research trends concerning immune response in this context remain limited. To address this knowledge gap, this bibliometric analysis aims to provide valuable insights that can inform future advancements in understanding immune response to human brucellosis, offering a thorough overview of the current state of research in this field.

## Materials and methods

### Data sources and search strategy

The literature search was conducted utilizing the Web of Science Core collection (WoSCC) to retrieve pertinent articles published between January 1980 and September 2024. The search strategy was delineated below: ((TS = (brucella∗)) AND TS = (“human*” OR “people*” OR “person*” OR “man” OR “mankind*” OR “patient*”)) AND TS = (“Immune response” OR “immune system” OR “immunological system” OR “innate immunity” OR “adaptive immunity” OR “antigen-presenting cells” OR “T cell*” OR “B cell*” OR “natural killer cell*” OR “macrophage*” OR “dendritic cell*” OR “Th1” OR “Th17” OR “Treg”) (Ferrero et al., 2020; Golding et al., 2001; Martirosyan and Gorvel, 2013). This study concentrated exclusively on articles published in English. Bibliographic data, which included publication year, country or region, institution, journal, authorship details, and keywords, were exported in both “Full record and cited references” and “plain text” formats for bibliometric analysis (Appendix).

### Data analysis and visualization

This study employed VOSviewer 1.6.20 and CiteSpace 6.3 for visualization analysis, in conjunction with R 4.3.3. VOSviewer is a multifunctional software tool that significantly aided in mapping co-occurrence and coupling of journals, institutional collaborations, co-occurrence networks of author, and co-occurrence analysis of keywords. It facilitates the visualization and exploration of intricate relationships within academic disciplines, offering insights into co-authorship, co-citation, and keyword co-occurrence networks (Arruda et al., 2022). In these visualizations, node size corresponded to the volume of publications, line thickness indicated the strength of relationships, and node color signified publication time, thereby illuminating significant research trends over time (Liu et al., 2022). In contrast, CiteSpace focused on identifying keyword bursts (Byndloss and Tsolis, 2016), with time slicing set from January 1980 to September 2024, using one-year intervals. The node type was designated as keywords, with a threshold of the top five keywords per slice and pruning implemented using Pathfinder combined with merged network pruning. Based on the parameter settings for each node, visualization analysis was performed to generate a keyword timeline map for the research field of human brucellosis immune response. In the trend visualization, blue lines represented the duration of citations, while red segments indicated the duration of keyword bursts. R 4.3.3 is employed for trend mapping and ranking analysis, enabling the tracking of publication and citation patterns among authors, institutions, and countries. This tool also supports the generation of trend maps and longitudinal analyses pertaining to developments within the research field (Zhao and Li, 2023). Bibliometric indicators, including the *h*-index, *g*-index, and *m*-index, were utilized to evaluate the academic impact of authors and journals. The *h*-index quantified productivity and citation impact, the *g*-index emphasized the importance of highly cited papers, and the *m*-index reflected the growth rate of the *h*-index over time (Thompson et al., 2009). These metrics are instrumental in assessing the relative influence of researchers and journals within the field. Additionally, the Journal Citation Reports (JCR) classifies journals based on impact factor (IF), which measures the average number of citations per article published in each journal (Cummins and Serruys, 2016), thereby providing insights into their relative significance within the discipline.

## Results

### Overview of publication status

**Figure 1:**
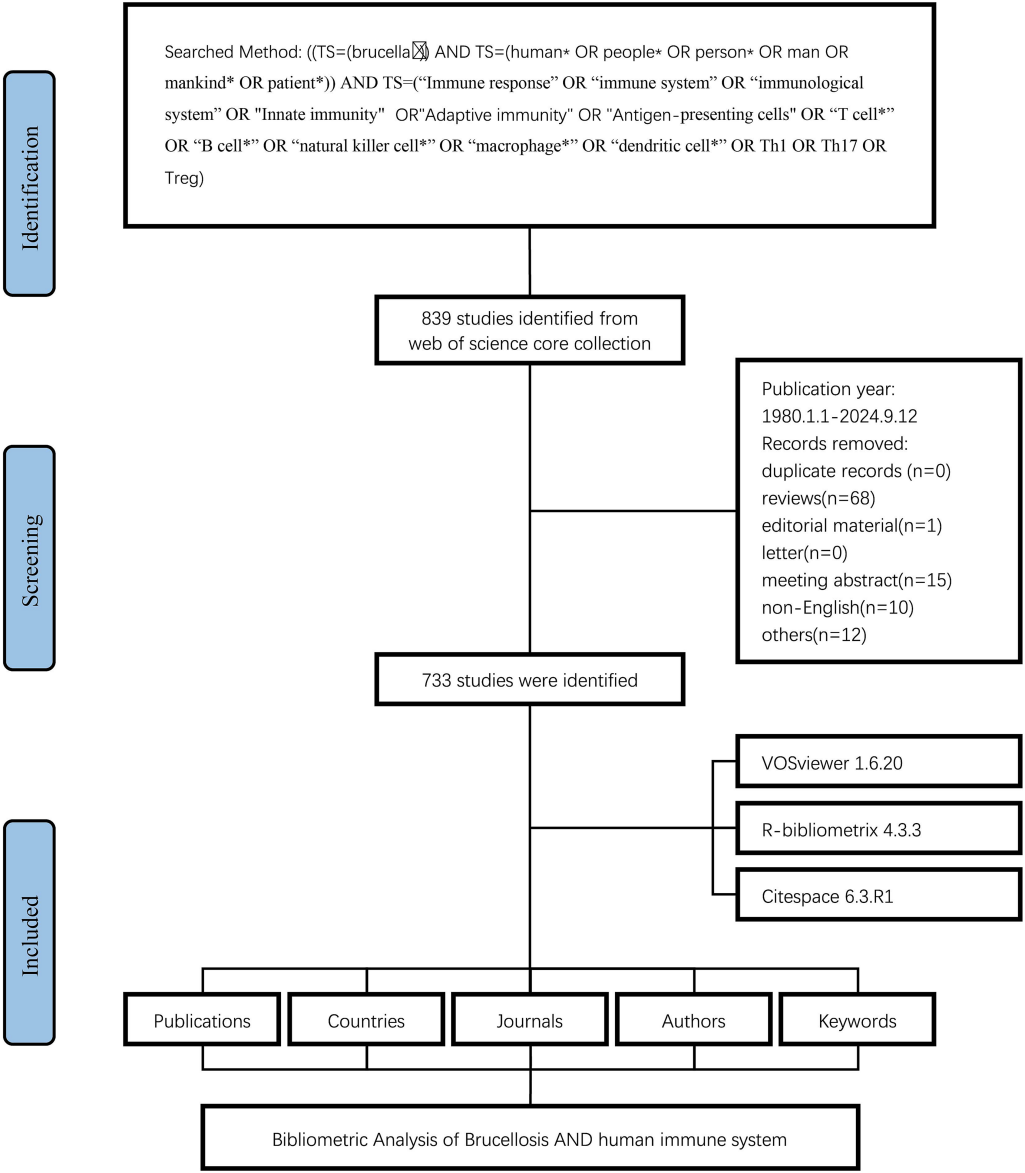
Flowchart of data screening process.

The flowchart of the study was depicted in Figure 1. Between 1980 and 2024, a total of 733 articles addressing the immune response to human brucellosis were identified. This analysis indicated that 3,251 authors from 1,918 institutions across 49 countries contributed to these manuscripts. These works were disseminated across 243 journals and cited a total of 19,690 sources, yielding an average of 29.21 citations per article.

### Analysis of annual publication trend

**Figure 2:**
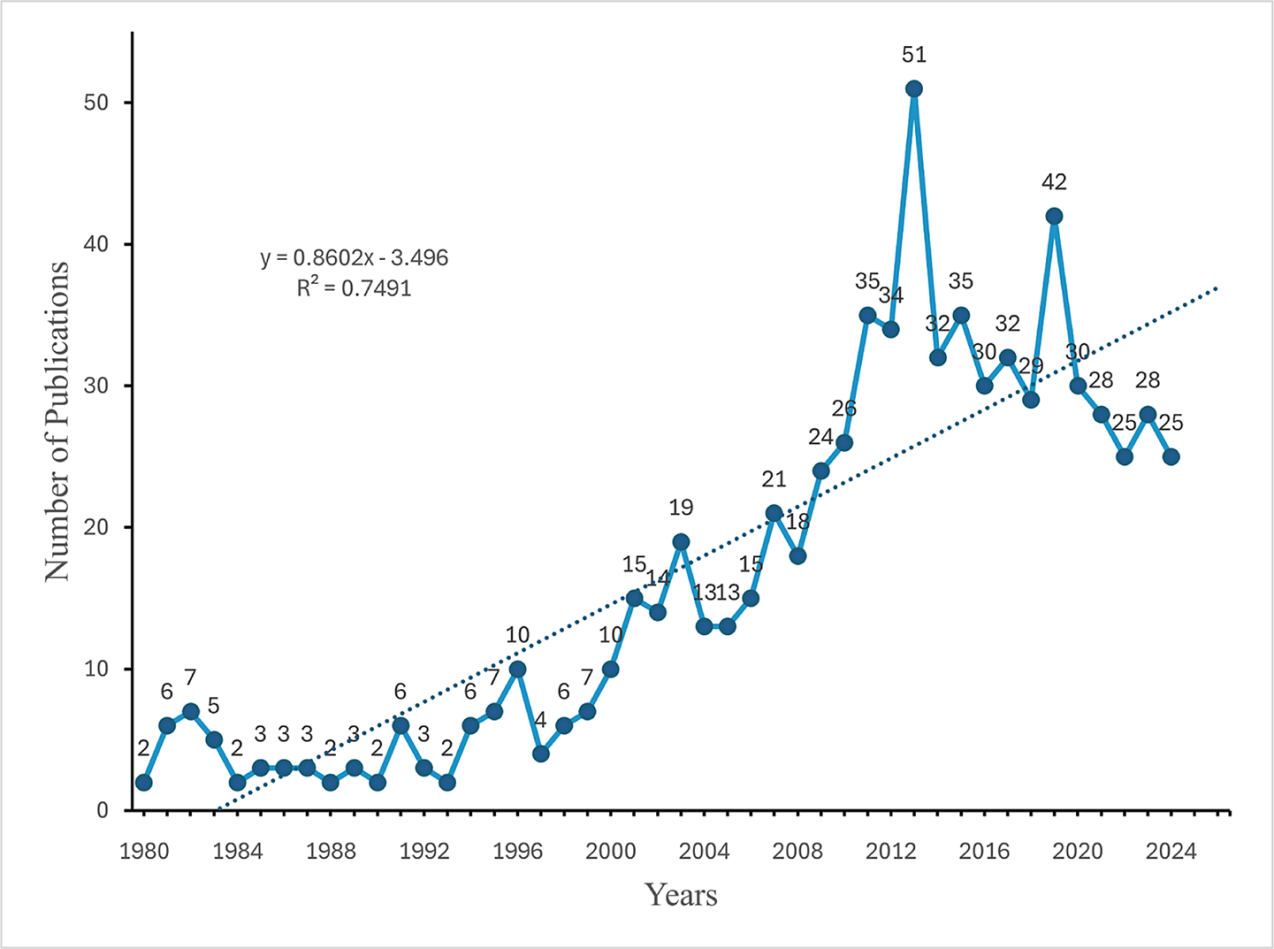
Annual growth of publications on immune response to human brucellosis from 1980 to 2024.

To comprehend the evolution of research within this domain, an analysis of annual publication trends was conducted. From 1980 to the mid-1990s, fewer than 10 articles were published annually. Beginning in 1996, the volume of publications experienced a significant increase, culminating in a peak of 51 publications in 2013. Following this peak, the number of publications exhibited fluctuations, reaching another high of 42 publications in 2020 (Figure 2).

### Analysis of countries

**Figure 3:**
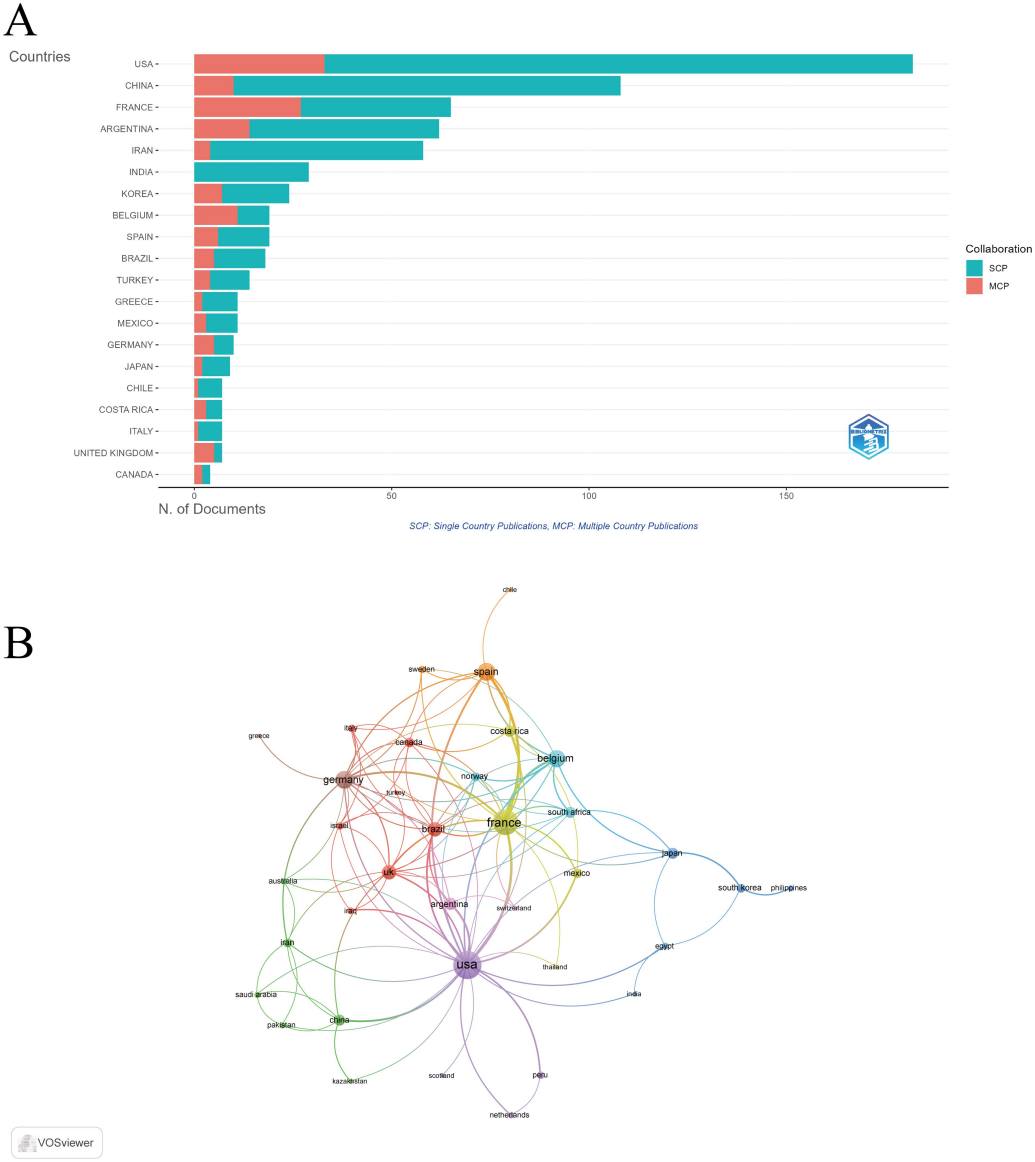
Global distribution and collaboration in immune response to human brucellosis. **(A)** Distribution of corresponding authors’ publications by country. **(B)** Visualization map depicting the collaboration. Nodes are countries, sized by publication count. Links show co-occurrence, with thickness indicating collaboration strength. Colors represent research clusters. Link strength reflects co-occurrence frequency.

The bibliometric analysis indicated that publications on this subject were produced by 49 distinct countries and regions. The United States emerged as the leader with 182 publications, representing 24.8% of the total output, followed by China (108 publications, 14.7%), France (65 publications, 8.9%), Argentina (62 publications, 8.5%), and Iran (58 publications, 7.9%). In terms of citation impact, the United States garnered the highest total citation count at 7,369 citations, followed by France with 3,850 citations and Argentina with 1,561 citations. Notably, France also exhibited the highest average citations per publication, recorded at 59.2, whereas the average for publications from China was significantly lower at 9.3 (Supplementary Table S1). Additionally, the United States led in the number of publications involving international collaboration, succeeded by France and Argentina (Figure 3A). Furthermore, the collaboration among countries was visualized using VOSviewer. As shown in Figure 3B, the United States, France, and Germany were the top three countries with the strongest international collaboration network.

### Analysis of institutions

**Figure 4:**
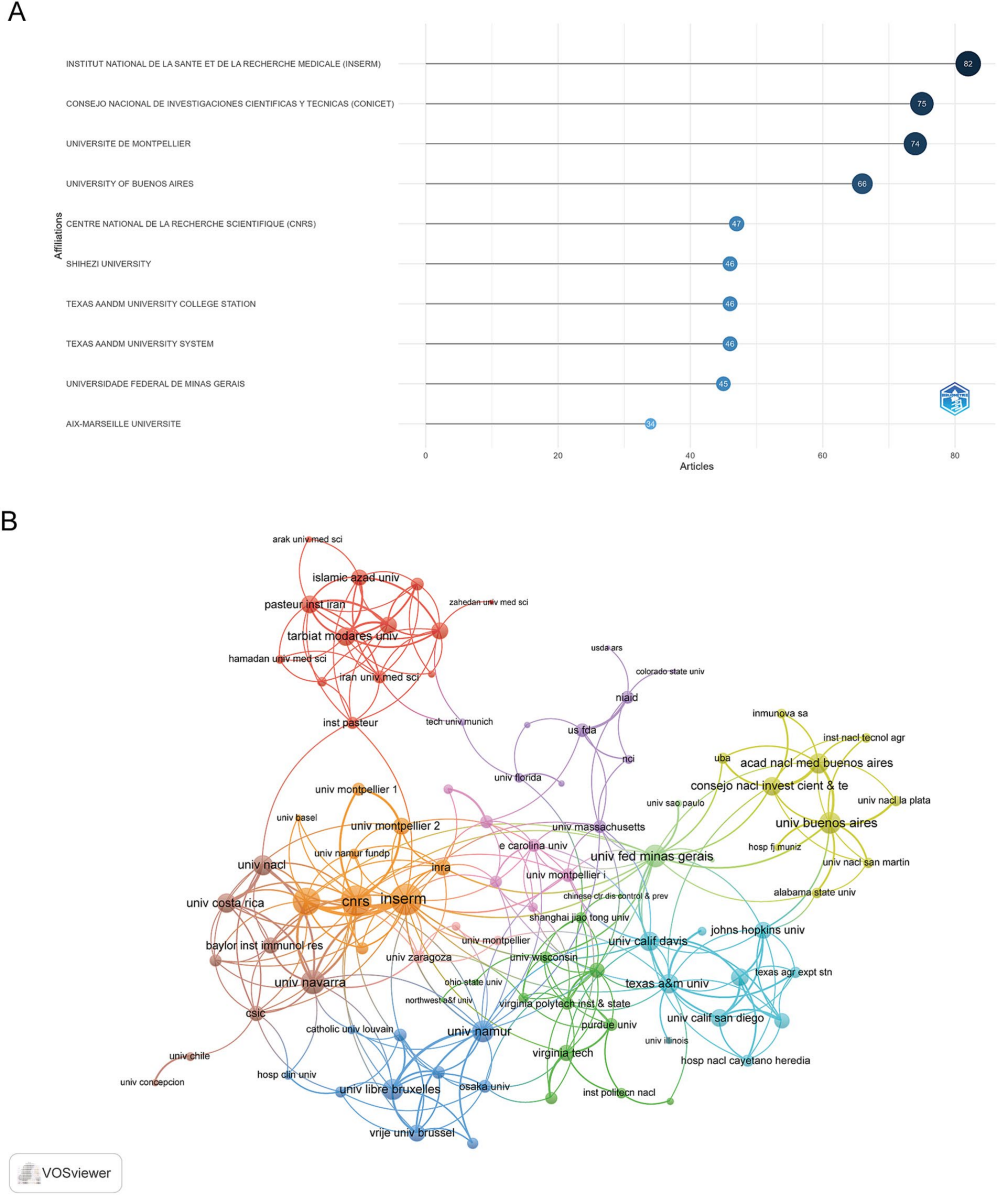
Institutional contributions and collaborations in immune response to human brucellosis. **(A)** Top 10 institutions by article count and rank. **(B)** Visualization map depicting the collaboration among different institutions. Nodes are institutions, sized by publication count. Links show co-occurrence, with thickness indicating collaboration strength. Colors represent research clusters. Link strength reflects co-occurrence frequency.

A total of 1,918 institutions engaged in research within this field, and their publication outputs were systematically analyzed. The 10 most productive institutions were presented in Figure 4A. The Institut National de la Santé et de la Recherche Médicale (INSERM) led in research output, contributing 82 articles, followed by the Consejo Nacional de Investigaciones Científicas y Técnicas (CONICET) with 75 articles, and the Université de Montpellier with 74 articles. VOSviewer was employed to visualize collaboration among these institutions, as illustrated in Figure 4B. INSERM exhibited the largest node, indicating the highest level of collaboration with other institutions, followed by the Centre National de la Recherche Scientifique (CNRS) and Aix Marseille Université.

### Analysis of authors

**Figure 5:**
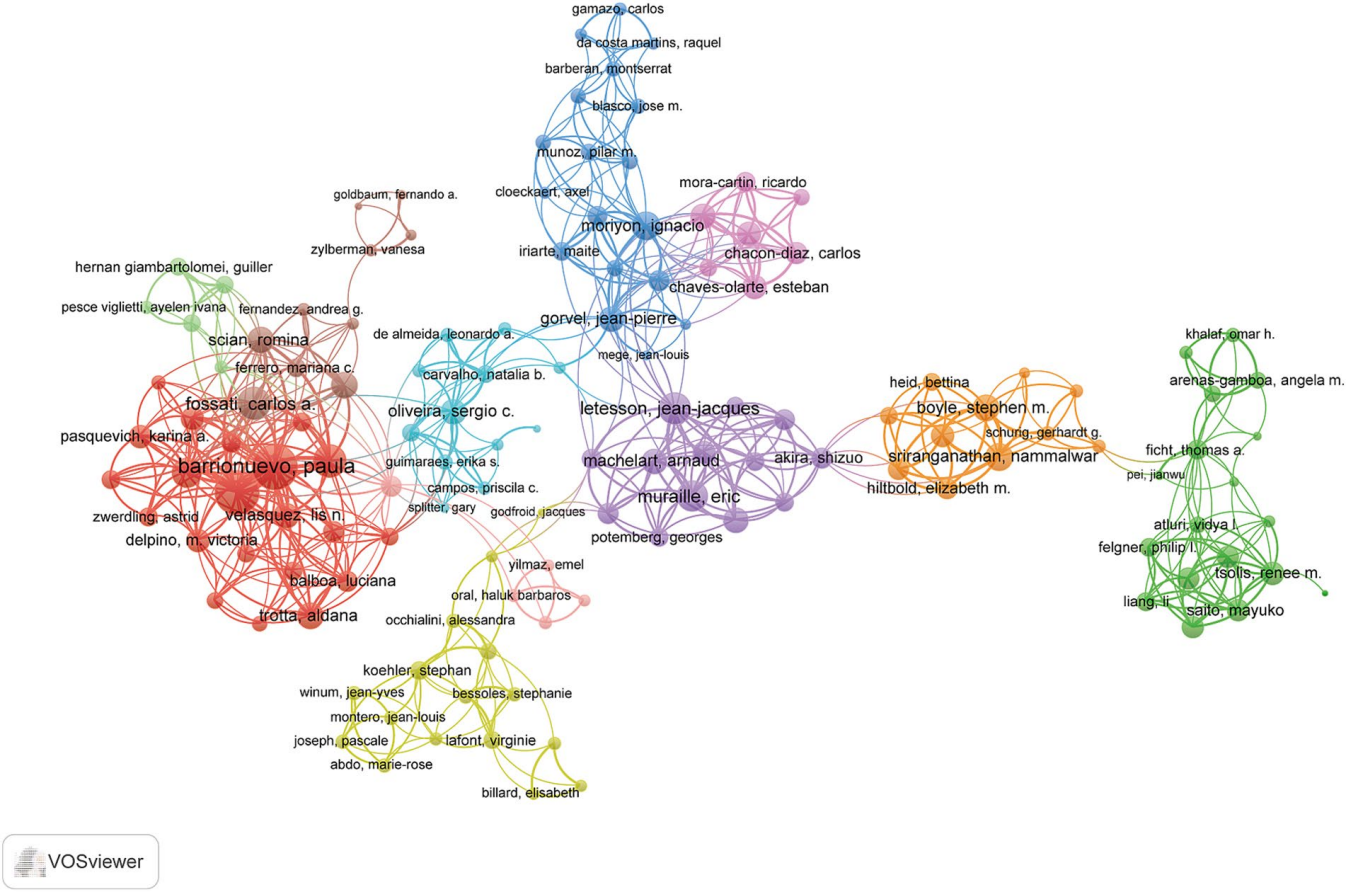
Author collaboration network in immune response to human brucellosis. Nodes are authors, sized by publication count. Links show co-occurrences, with thickness indicating collaboration strength. Colors represent research clusters. Link strength reflects co-occurrence frequency.

A total of 3,251 authors contributed to this field of study. The authors were ranked according to their *h*-index to identify high-impact researchers (Supplementary Table S2). Liautard J. P. ranked first in both total publications and total citations, producing 23 articles and accumulating 1,521 citations. Furthermore, Liautard J. P. attained the highest *h*-index, with a score of 20. A collaborative network analysis of authors with three or more publications was performed using VOSviewer. Among the 136 authors involved in international collaborations. Barrionuevo, Paula had the highest number of collaborations with other authors, followed by Giambartolomei, Guillermo H. and Chen, Chuangfu (Figure 5).

### Analysis of journals

**Figure 6:**
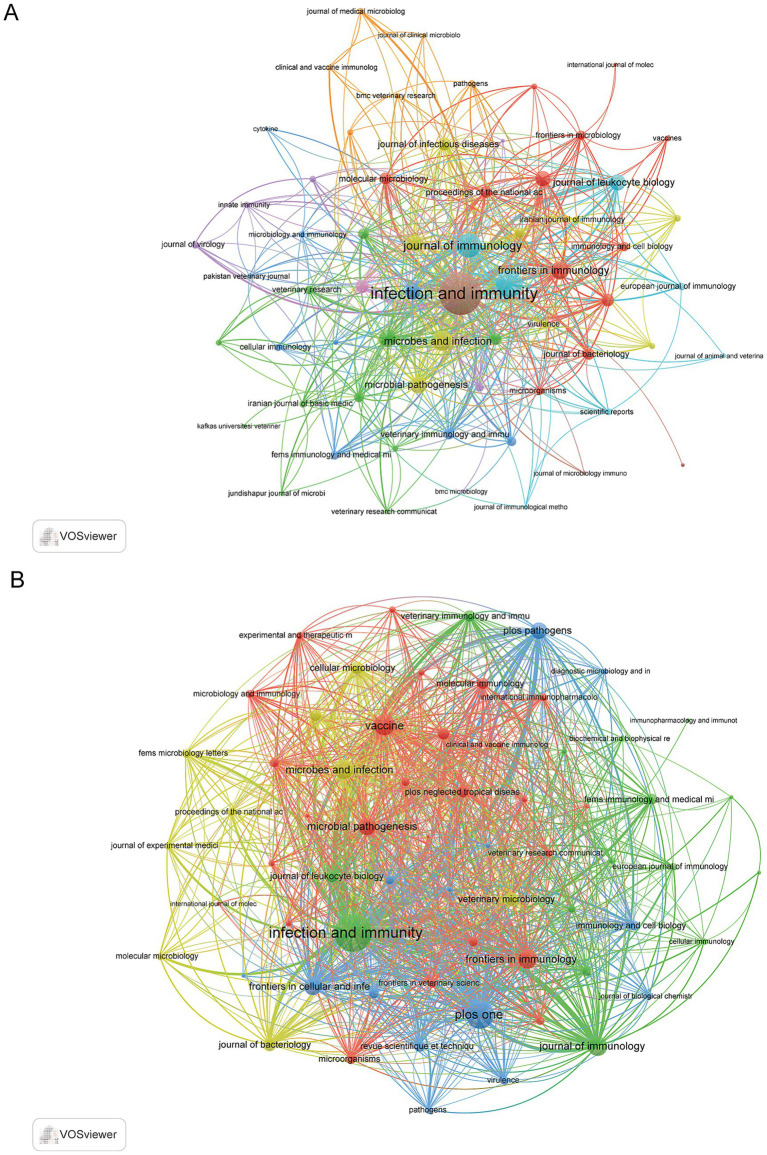
Network analyses of journals in immune response to human brucellosis. **(A)** The co-occurrence networks of journals. Nodes are journals, sized by article count. Links show co-citation in articles, with thickness indicating strength. Colors represent thematic clusters. **(B)** The coupling networks of journals. Nodes are journals, sized by article count. Links show co-citation in articles, with thickness indicating strength. Colors represent thematic clusters.

The bibliometric analysis indicated that the research was disseminated through 243 distinct journals. *Infection and Immunity* stood out as the most productive journal, with 97 documents, demonstrating its central role in the field, followed by *PLoS One* with 39 documents and *Vaccine* with 31. Furthermore, *Infection and Immunity* was the most cited journal with 3,861 citations, followed by *Journal of Immunology* (1,660) and *Vaccine* (724) (Supplementary Table S3). Co-occurrence network analysis revealed that the three principal journals with the highest total link strength were *Infection and Immunity* (792), *PLoS One* (299), and *Journal of Immunology* (261) (Figure 6A). Furthermore, coupling network analysis demonstrated that the same three journals—*Infection and Immunity* (20,520), *PLoS One* (10,769), and the *Journal of Immunology* (6,496), also exhibited the highest total link strength (Figure 6B).

### Analysis of research hotspots and frontiers

**Figure 7:**
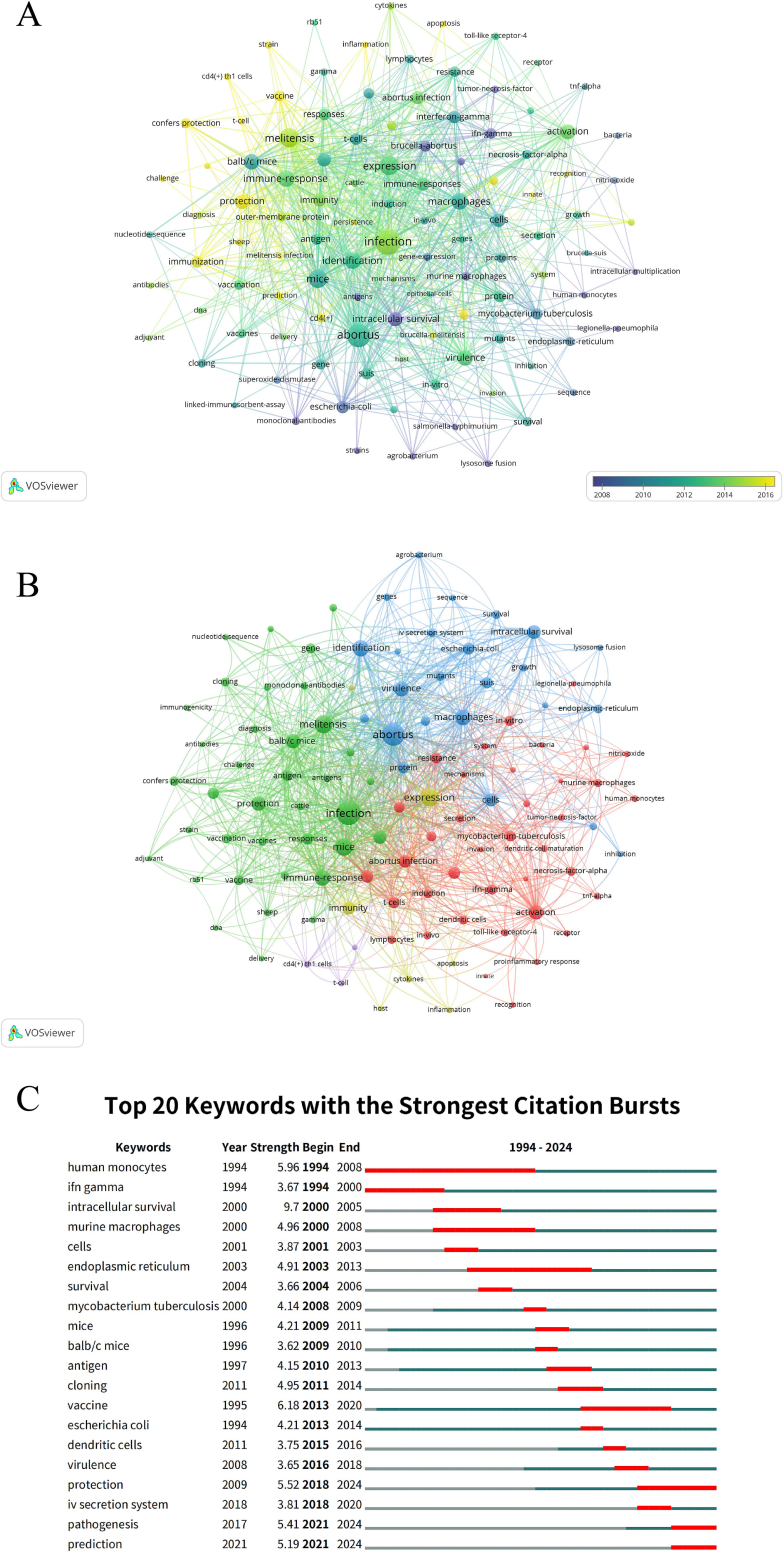
Keyword analysis in immune response to human brucellosis. **(A)** Visual analysis of keyword co-occurrence network (time trend). Nodes are keywords, sized by frequency. Links show co-occurrence in articles, with thickness indicating strength. Colors reflect average publication year (see color gradient). **(B)** Keyword co-occurrence network (clusters). Nodes are keywords, sized by frequency. Links show co-occurrence in articles, with thickness indicating strength. Colors represent distinct research clusters based on thematic similarities. **(C)** Top 20 keywords with the strongest citation bursts. In the trend visualization, the blue line shows the duration of citations, and the red segment indicates the duration of citation bursts.

The study identified 109 keywords, each appearing at least 10 times. Co-occurrence network analysis revealed that “infection,” “melitensis,” “abortus,” “identification,” and “expression” were the most frequently co-occurring keywords (Supplementary Table S4). From 2008 to 2016, keyword evolution occurred in three phases: the early phase (2008–2010) focused on basic pathogens (e.g., “escherichia-coli”) and cellular mechanisms (e.g., “lysosome-fusion”), emphasizing antibody techniques and immunological detection; the middle phase (2010–2012) shifted to keywords such as “infection,” “immune-responses,” and “macrophages,” with research exploring infection mechanisms, *in vivo* experiments, and specific pathogens like “brucella-abortus”; the recent phase (2014–2016) highlighted keywords like “toll-like-receptor-4,” “inflammation,” and “vaccine,” focusing on immune signaling pathways, inflammatory response, and vaccine development, reflecting a shift from basic to applied research (Figure 7A). The co-occurrence network identified three main clusters: the green cluster centered on *Brucella* vaccine development and immune protection, with key terms including “infection,” “protection,” “antigen,” “antibodies,” “vaccine/vaccination,” “melitensis,” and “balb/c mice”; the blue cluster focused on *Brucella* molecular mechanisms and intracellular survival strategies, with key terms such as “abortus,” “intracellular survival,” “virulence,” “type IV secretion system,” “macrophages,” and “endoplasmic-reticulum”; the red cluster addressed host innate immunity and *Brucella* interaction mechanisms, with core keywords including “dendritic cells,” “activation,” “tnf-alpha,” “innate,” and “proinflammatory response” (Figure 7B and Supplementary Table S5). Burst analysis of keywords showed that among the top 20 keywords with the highest citation counts sustained over at least 1 year, “vaccine” had the highest citation burst strength (strength = 6.18). Notably, since 2018, keywords such as “protection,” “type IV secretion system,” “pathogenesis,” and “prediction” gained significant attention (Figure 7C).

## Discussion

### Overview of the main findings

This bibliometric analysis investigated the research landscape of immune response to human brucellosis from 1980 to 2024, analyzing 733 publications from diverse countries, institutions, and authors. A significant finding was that *Brucella*-related studies primarily centered on vaccine development and immune protection, molecular mechanisms and intracellular survival strategies of *Brucella*, and interactions between host innate immunity and *Brucella*. Mechanistic studies and vaccine development would continue to be central trends in future research.

The United States emerged as the predominant country in terms of both publication output and citation impact, consistent with its leadership in global health research. Additionally, China, France, and Argentina have made significant contributions to the study of brucellosis. The disease remains prevalent in regions such as the Middle East, the Mediterranean, Central and South America, and East Asia (Pappas et al., 2006). Consequently, institutions such as INSERM in France and CONICET in Argentina were among the most prolific, reflecting high proportion of research and development budget, reflecting a substantial proportion of research and development funding. Furthermore, as a country with a significant agricultural industry, China faces a critical situation regarding brucellosis prevention and control (Deqiu et al., 2002). The incidence of human brucellosis cases is increasing, and the affected regions are gradually expanding from the north to the south, with the northern regions, particularly Inner Mongolia, identified as high-incidence areas (Wang et al., 2013). This information is valuable for researchers seeking to identify key regions and institutions that are at the forefront of this field. However, the average citation frequency per paper in China was only 9.3, indicating that Chinese authors experience lower citation rates, which underscores the necessity for the publication of high-quality research. International collaborations were pivotal in amplifying research dissemination, fostering access to diverse networks, increasing visibility, and promoting innovative idea exchange across global scientific communities. These findings are valuable for researchers aiming to identify leading regions and institutions in brucellosis research.

### Hotspots and frontiers

Keyword co-occurrence and burst detection analysis provided valuable insights into thematic development and research trends organized into three clusters: vaccine development molecular mechanisms and host innate immunity. These clusters reflected the evolution of research from understanding fundamental immune response to addressing specific challenges in vaccine design and therapeutic interventions.

#### Green cluster: *Brucella* vaccine development and immune protection

Brucellosis’s zoonotic nature necessitates effective vaccines to protect both human and animal populations, as it threatens public health and livestock economies (Abushahba et al., 2024). The Th1 immune response, driven by antigens like lipopolysaccharide (LPS) and outer membrane proteins (OMPs), is central to protection, promoting cellular immunity critical for controlling intracellular *Brucella* (Khoramabadi et al., 2025). Existing animal vaccines (e.g., S19, RB51, Rev.1) achieve ~70% efficacy but face challenges such as human infection risk, spontaneous abortion, antibiotic resistance, and limited protection (Abushahba et al., 2024; Molina et al., 2024; de Oliveira et al., 2022). Recent advances include novel vaccine strategies. For instance, Molina et al. (2024) developed a DNA vaccine using a DNA prime-protein boost approach, enhancing immunogenicity with *Brucella* antigens, though further optimization is needed. Another study introduced rMEV-Fc, a polypeptide vaccine incorporating OMP19, OMP16, OMP25, and L7/L12 antigens, showing promise in animal models (Wu et al., 2025). A novel finding highlighted BspF-mediated crotonylation at TRIM38’s K142 site, regulating NF-κB and MAPK pathways to modulate inflammation, offering insights for vaccine adjuvant design (Zhang H. et al., 2025). However, reliance on BALB/c mouse models limits clinical translation due to differences in human immune response, necessitating validation in large animal models like sheep. Future research should focus on optimizing vaccine strains and immunization strategies tailored to high-prevalence regions, such as the Mediterranean, to accelerate clinical application.

#### Blue cluster: *Brucella* molecular mechanisms and intracellular survival strategies

*Brucella*’s success as a pathogen stems from its ability to evade host immunity through virulence factors like LPS, type IV secretion system (T4SS), and OMPs, enabling intracellular survival (Zhang M. et al., 2025). Research on *Brucella abortus* and melitensis prioritizes identifying these factors to uncover therapeutic targets. For example, OMP16, a pathogen-associated molecular pattern, interacts with TLR4, and the anti-OMP16 IgM monoclonal antibody B7 agglutinates *Brucella abortus* A19, activating complement-mediated bactericidal effects (Zhai et al., 2025). Additionally, A19 infection upregulates SIRT2, inducing mitochondrial apoptosis, while SIRT2 inhibition reduces bacterial survival, suggesting a novel therapeutic avenue (Zhang M. et al., 2025). The ArsR6 transcription factor also regulates host cell death (Zhu et al., 2025). Despite progress, clinical translation remains challenging. Future efforts could develop T4SS inhibitors or target endoplasmic reticulum pathways to disrupt *Brucella*’s survival, paving the way for new treatments and vaccines.

#### Red cluster: host innate immunity and *Brucella* interaction mechanisms

The innate immune system, involving dendritic cells, macrophages, and pro-inflammatory cytokines, forms the first line of defense against *Brucella*, shaping subsequent adaptive responses (Miraglia et al., 2013). Studies showed *Brucella abortus* induces MMP-9 production in astrocytes via MAPK signaling, suggesting MAPK inhibitors as potential therapies for neurobrucellosis (Miraglia et al., 2013). Recombinant proteins rOMP10, rOMP19, and rBP26 promote bone marrow-derived dendritic cell (BMDC) maturation, upregulate TLR-2/4, and enhance pro-inflammatory cytokines (TNF-α, IFN-γ, IL-6, IL-12), stimulating T-cell proliferation, while rOMP25 and rOMP31 suppress BMDC maturation and promote anti-inflammatory cytokines (IL-10, IL-4) (Xu et al., 2024). A meta-analysis identified T-cell dysfunction in human brucellosis, with altered CD4^+^/CD8^+^ ratios driving disease progression (Zheng et al., 2018). *Brucella canis* in canine macrophages upregulates TLR-3/7/8 and pro-inflammatory cytokines, highlighting TLR-mediated immunity’s role (Bin Park et al., 2023). Genetic polymorphisms, such as IL-4 rs2243250 and IL-18 rs1946519, increase brucellosis risk, while others (e.g., IFN-γ UTR5644) are protective (Zafari et al., 2020). Ultra-long-chain fatty acids in *Brucella* LPS induce immunosuppression, but Bm-ΔBacA LPS promotes inflammation, offering targets for immune modulation (Zhang J. D. et al., 2025). Future strategies targeting MAPK or TLR pathways could optimize innate immunity, supporting novel adjunctive therapies.

### Emerging research directions

Citation bursts for keywords “protection,” “type IV secretion system” (T4SS), “pathogenesis,” “vaccine,” and “prediction” highlight recent hotspots in human brucellosis immune response research. *Brucella*’s immune escape strategies, such as suppressing inflammation and modulating T-cell function, drive chronic infection, prompting focus on vaccines, molecular mechanisms, and predictive tools (Scott and Jackson, 2015).

The “Vaccine” (2013–2020) showed the strongest citation burst, underscoring its pivotal role in brucellosis control. Vaccines shape immune memory, reducing infectious disease burden, and are deemed the most effective strategy by the World Health Organization (Iwasaki and Omer, 2020). Attenuated live vaccines (e.g., *Brucella abortus* A19, *Brucella suis* S2, *Brucella melitensis* M5, Rev.1) are effective in animals but pose risks, including miscarriage and human infection (Baoshan et al., 2021), rendering them unsuitable for human use (Perkins et al., 2010). Advances in genomics, recombinant DNA technology, and bioinformatics enable engineered vaccines, yet safety and efficacy require further study (DelVecchio et al., 2002). Future research should prioritize safe human vaccines, leveraging multi-omics (e.g., transcriptomics, proteomics) to optimize antigens for high-prevalence regions like the Mediterranean.

Keyword “Type IV secretion system” (2018–2020) was a key research focus. T4SS modulates host immune pathways (e.g., NF-κB) promoting *Brucella* survival and persistent infection (Zheng et al., 2024). Its assembly driven by signals like low pH and regulators (e.g., LuxR-like histidine utilization) remains underexplored (Ke et al., 2015). Future studies should elucidate T4SS regulatory mechanisms and develop inhibitors to disrupt *Brucella*’s intracellular survival informing novel therapies and vaccines.

The term “Prediction” (2021–2024) reflects growing reliance on predictive models for immune response and infection outcomes. Brucellosis management requires prolonged, unique antimicrobial regimens, necessitating timely diagnosis via culture, serology, or molecular methods (Di Bonaventura et al., 2021; Elbehiry et al., 2023). The SARIMAX model excels in predicting geographic disease patterns, guiding early diagnosis in high-risk areas (Chen et al., 2023). Future efforts should integrate multi-omics and machine learning to build precise predictive models, enhancing diagnosis, control strategies, and personalized treatments linked to immune escape mechanisms.

These trends position immune escape mechanisms, vaccine development, and predictive modeling as research frontiers. Future studies should advance safe vaccines, T4SS-targeted therapies, and data-driven prediction to address chronic brucellosis challenges.

### Limitations

This study presented several limitations. First, while it included a broad range of publications, it might have underrepresented significant studies, particularly those published in smaller, regional, or non-English journals due to the indexing limitations of the WoSCC. While our dataset provided a robust overview of global high-impact research, it might not have fully captured contributions from low- and middle-income countries (LMICs) or other underrepresented regions. Second, the data analysis relied predominantly on a single database, which may have been subject to the biases associated with the selection of data sources, potentially omitting valuable research that lay outside the indexed scope. Furthermore, although the findings of this study were current, they were subject to rapid change in the dynamic scientific field, underscoring the necessity for ongoing analysis to identify emerging trends.

This study conducts a bibliometric analysis to reveal the research trend of immune response to human brucellosis. Identification of immune escape mechanisms is the current focus of the field, and the development of safe and effective vaccines for human brucellosis is a future concern.

## Data Availability

The original contributions presented in the study are included in the article/Supplementary material, further inquiries can be directed to the corresponding author.
