# Supplementary material for: Mapping the research landscape of immune response in human brucellosis: a bibliometric analysis
Source: Front Microbiol. 2025 Jul 29;16:1583520. doi: 10.3389/fmicb.2025.1583520 (PMC12339437; doi:10.3389/fmicb.2025.1583520)
Supplement: Supplementary file 1 [file Data_Sheet_1.docx]

**Table S1.** **Publication and citation profiles of leading countries.**

| **Country** | **Articles** | **Freq** | **MCP_Ratio** | **TP** | **TP_rank** | **TC** | **TC_rank** | **Average Citations** |
| --- | --- | --- | --- | --- | --- | --- | --- | --- |
| USA | 182 | 0.248 | 0.181 | 486 | 1 | 7369 | 1 | 40.5 |
| China | 108 | 0.147 | 0.093 | 351 | 2 | 1002 | 4 | 9.3 |
| France | 65 | 0.089 | 0.415 | 173 | 4 | 3850 | 2 | 59.2 |
| Argentina | 62 | 0.085 | 0.226 | 161 | 5 | 1561 | 3 | 25.2 |
| Iran | 58 | 0.079 | 0.069 | 208 | 3 | 715 | 6 | 12.3 |
| India | 29 | 0.040 | 0.000 | 70 | 9 | 588 | 9 | 20.3 |
| Korea | 24 | 0.033 | 0.292 | 71 | 8 | 291 | 13 | 12.1 |
| Belgium | 19 | 0.026 | 0.579 | 73 | 7 | 862 | 5 | 45.4 |
| Spain | 19 | 0.026 | 0.316 | 87 | 6 | 597 | 8 | 31.4 |
| Brazil | 18 | 0.025 | 0.278 | 61 | 10 | 627 | 7 | 34.8 |
| Turkey | 14 | 0.019 | 0.286 | 42 | 13 | 127 | 20 | 9.1 |
| Greece | 11 | 0.015 | 0.182 | 29 | 15 | 227 | 16 | 20.6 |
| Mexico | 11 | 0.015 | 0.273 | 50 | 12 | 145 | 18 | 13.2 |
| Germany | 10 | 0.014 | 0.500 | 51 | 11 | 513 | 10 | 51.3 |
| Japan | 9 | 0.012 | 0.222 | 27 | 16 | 415 | 11 | 46.1 |
| Chile | 7 | 0.010 | 0.143 | 17 | 19 | 83 | 21 | 11.9 |
| Costa Rica | 7 | 0.010 | 0.429 | 29 | 14 | 374 | 12 | 53.4 |
| Italy | 7 | 0.010 | 0.143 | 25 | 17 | 149 | 17 | 21.3 |
| United Kingdom | 7 | 0.010 | 0.714 | 25 | 18 | 240 | 15 | 34.3 |
| Canada | 4 | 0.005 | 0.500 | 11 | 23 | 143 | 19 | 35.8 |

Note(s): Articles: Publications of Corresponding Authors only. Freq: Frequence of Total Publications. MCP_Ratio: Proportion of Multiple Country Publications. TP: Total Publications. TP_rank: Rank of Total Publications. TC: Total Citations. TC_rank: Rank of Total Citations. Average Citations: The average number of citations per publication.

**Table S2. Publication and citation profiles of high-impact authors**

| **Authors** | **H_index** | **g-index** | **m-index** | **PY_start** | **TP** | **TP_Frac** | **TP_rank** | **TC** | **TC_rank** |
| --- | --- | --- | --- | --- | --- | --- | --- | --- | --- |
| Liautard JP | 20 | 23 | 0.65 | 1994 | 23 | 4.45 | 1 | 1521 | 1 |
| Barrionuevo Paula | 15 | 22 | 0.88 | 2008 | 22 | 2.61 | 2 | 657 | 6 |
| Dornand J | 15 | 15 | 0.48 | 1994 | 15 | 2.73 | 7 | 1109 | 2 |
| Fossati Carlos A. | 15 | 16 | 0.88 | 2008 | 16 | 3.07 | 6 | 666 | 5 |
| Giambartolomei Guillermo H. | 15 | 21 | 0.88 | 2008 | 21 | 2.53 | 3 | 670 | 4 |
| Gorvel Jean-Pierre | 13 | 14 | 0.77 | 2008 | 14 | 2.51 | 8 | 643 | 8 |
| Baldi Pablo C. | 12 | 13 | 0.75 | 2009 | 13 | 2.71 | 11 | 395 | 18 |
| Victoria Delpino M. | 12 | 13 | 0.80 | 2010 | 13 | 1.77 | 13 | 380 | 19 |
| Golding B | 11 | 17 | 0.26 | 1982 | 17 | 2.83 | 5 | 459 | 12 |
| Moriyon Ignacio | 11 | 12 | 0.61 | 2007 | 12 | 1.27 | 15 | 656 | 7 |
| Oliveira Sergio C. | 11 | 13 | 0.73 | 2010 | 13 | 2.06 | 12 | 406 | 17 |
| Gross A | 10 | 10 | 0.35 | 1996 | 10 | 1.97 | 22 | 757 | 3 |
| Sriranganathan Nammalwar | 10 | 14 | 0.53 | 2006 | 14 | 2.37 | 9 | 312 | 25 |
| Boyle Stephen M. | 9 | 11 | 0.47 | 2006 | 11 | 1.42 | 16 | 256 | 28 |
| Cassataro Juliana | 9 | 10 | 0.53 | 2008 | 10 | 1.19 | 20 | 379 | 20 |
| Letesson Jean-Jacques | 9 | 12 | 0.50 | 2007 | 12 | 1.40 | 14 | 634 | 9 |
| Muraille Eric | 9 | 11 | 0.50 | 2007 | 11 | 1.16 | 19 | 434 | 15 |
| Moreno Edgardo | 8 | 9 | 0.44 | 2007 | 9 | 1.02 | 26 | 444 | 14 |
| Scian Romina | 8 | 8 | 0.53 | 2010 | 8 | 1.12 | 32 | 250 | 30 |
| Zhang Hui | 8 | 12 | 0.67 | 2013 | 14 | 1.51 | 10 | 162 | 37 |

Note(s): H_index: The h-index of the journal, which measures both the productivity and citation impact of the publications. g_index: The g-index of the journal, which gives more weight to highly-cited articles. m_index: The m-index of the journal, which is the h-index divided by the number of years since the first published paper. TP: Total Publications. TP_rank: Rank of Total Publications. TC: Total Citations. TC_rank: Rank of Total Citations. Average Citations: The average number of citations per publication. PY_start: Publication Year Start, indicating the year the journal started publication.

**Table S3. Bibliometric Indicators of High-Impact Journals**

| **Journal** | **H_index** | **IF** | **JCR_Quartile** | **PY_start** | **TP** | **TP_rank** | **TC** | **TC_rank** |
| --- | --- | --- | --- | --- | --- | --- | --- | --- |
| Infection and Immunity | 43 | 2.9 | Q2 | 1981 | 97 | 1 | 3861 | 1 |
| Plos One | 22 | 2.9 | Q1 | 2007 | 39 | 2 | 648 | 7 |
| Journal of Immunology | 19 | 3.6 | Q2 | 1982 | 30 | 4 | 1660 | 2 |
| Vaccine | 17 | 4.5 | Q2 | 1996 | 31 | 3 | 724 | 3 |
| Microbes and Infection | 12 | 2.6 | Q3 | 2003 | 17 | 6 | 428 | 10 |
| Microbial Pathogenesis | 10 | 3.3 | Q2 | 1997 | 17 | 7 | 232 | 22 |
| Plos Pathogens | 10 | 5.5 | Q1 | 2008 | 11 | 11 | 309 | 17 |
| Frontiers in Immunology | 9 | 5.7 | Q1 | 2017 | 17 | 5 | 202 | 23 |
| Journal of Bacteriology | 9 | 2.7 | Q3 | 1996 | 11 | 10 | 681 | 6 |
| Journal of Infectious Diseases | 8 | 5 | Q1 | 1985 | 8 | 14 | 314 | 16 |
| Veterinary Immunology and Immunopathology | 8 | 1.4 | Q2 | 1990 | 12 | 8 | 194 | 25 |
| Veterinary Microbiology | 8 | 2.4 | Q1 | 2002 | 9 | 13 | 714 | 4 |
| Cellular Microbiology | 7 | 2.6 | Q3 | 2004 | 7 | 16 | 405 | 11 |
| European Journal of Immunology | 7 | 4.5 | Q2 | 1980 | 7 | 18 | 318 | 15 |
| Frontiers in Cellular and Infection Microbiology | 7 | 4.6 | Q1 | 2011 | 11 | 9 | 187 | 26 |
| Journal of Leukocyte Biology | 7 | 3.6 | Q2 | 1994 | 7 | 19 | 242 | 21 |
| Molecular Immunology | 7 | 3.2 | Q3 | 2012 | 7 | 21 | 112 | 47 |
| Frontiers in Microbiology | 6 | 4 | Q2 | 2015 | 9 | 12 | 104 | 54 |
| Journal of Medical Microbiology | 6 | 2.4 | Q3 | 1998 | 7 | 20 | 138 | 40 |
| Clinical and Vaccine Immunology | 5 | N/A | N/A | 2011 | 5 | 25 | 105 | 52 |

Note(s): H_index: The h-index of the journal, which measures both the productivity and citation impact of the publications. IF: Impact Factor, indicating the average number of citations to recent articles published in the journal. JCR_Quartile: The quartile ranking of the journal in the Journal Citation Reports, indicating the journal's ranking relative to others in the same field (Q1: top 25%, Q2: 25%-50%, Q3: 50%-75%, Q4: bottom 25%). TP: Total Publications. TP_rank: Rank of Total Publications. TC: Total Citations. TC_rank: Rank of Total Citations. Average Citations: The average number of citations per publication. PY_start: Publication Year Start, indicating the year the journal started publication.

**Table S4. Top 20 keyword co-occurrence network analysis**

| **id** | **Keyword** | **Occurrences** | **Total link strength** |
| --- | --- | --- | --- |
| 883 | Infection | 165 | 741 |
| 24 | Abortus | 147 | 645 |
| 1121 | Melitensis | 90 | 432 |
| 592 | Expression | 97 | 416 |
| 1146 | Mice | 81 | 401 |
| 809 | Identification | 67 | 328 |
| 1085 | Macrophages | 71 | 320 |
| 836 | Immune-Response | 67 | 316 |
| 1890 | Virulence | 53 | 282 |
| 192 | Balb/C Mice | 56 | 274 |
| 1465 | Protection | 52 | 262 |
| 51 | Activation | 62 | 255 |
| 944 | Intracellular Survival | 50 | 236 |
| 1015 | Lipopolysaccharide | 47 | 225 |
| 323 | Cells | 56 | 222 |
| 838 | Immune-Responses | 49 | 200 |
| 26 | Abortus Infection | 43 | 200 |
| 840 | Immunity | 40 | 198 |
| 570 | Escherichia-Coli | 47 | 189 |
| 914 | Interferon-Gamma | 38 | 175 |

**Table S5.** Keyword Clustering Analysis

| **Cluster colour** | **Keywords** |
| --- | --- |
| Red | expression, innate, cells, secretion, mycobacterium tuberculosis, invasion, dendritic cell maturation, tumor necrosis factor, ifn-gamma, induction, tnf-alpha, activation, dendritic cells, toll-like receptor-4, receptor, necrosis factor-alpha, inhibition, proinflammatory response |
| Green | melitensis, infection, mice, challenge, antigen, vaccination, vaccines, responses, immun-response, immunity, sheep, dna, vaccine, adjuvant, strain, confers protection, cattle, antibodies, balb/c mice, diagnosis, immunogenicity, cloning, nucleotide sequence, gene, identification, monoclonal antibodies |
| Blue | abortus, virulence, macrophages in-vitro, growth, mutants, suis, legionella pneumophila, endoplasmic-reticulum, system, bacteria, nitric oxide, murine macrophages, human monocytes, resistance, protein, mechanisms, intracellular survival, survival, lysosome fusion, escherichia coli, iv secretion system, sequence, genes, type IV secretion system |
